# Diastolic function in heart transplant: From physiology to echocardiographic assessment and prognosis

**DOI:** 10.3389/fcvm.2022.969270

**Published:** 2022-10-31

**Authors:** Carlotta Sciaccaluga, Chiara Fusi, Federico Landra, Maria Barilli, Matteo Lisi, Giulia Elena Mandoli, Flavio D’Ascenzi, Marta Focardi, Serafina Valente, Matteo Cameli

**Affiliations:** ^1^Division of Cardiology, Department of Medical Biotechnologies, University of Siena, Siena, Italy; ^2^Division of Cardiology, Department of Cardiovascular Diseases - Azienda Unità Sanitaria Locale (AUSL) Romagna, “Santa Maria delle Croci” Hospital, Ravenna, Italy

**Keywords:** diastolic function, heart transplant, echocardiography, prognosis, physiology

## Abstract

Heart transplant (HTx) still represents the most effective therapy for end-stage heart failure, with a median survival time of 10 years. The transplanted heart shows peculiar physiology due to the profound alterations induced by the operation, which inevitably influences several echocardiographic parameters assessed during these patients’ follow-ups. With these premises, the diastolic function is one of the main aspects to take into consideration. The left atrium (LA) plays a key role in this matter, and that same chamber is significantly impaired with the transplant, with different degrees of altered function based on the surgical technique. Therefore, the traditional echocardiographic evaluation of diastolic function applied to the general population might not properly reflect the physiology of the graft. This review attempts to provide current evidence on diastolic function in HTx starting from defining its different physiology and how the standard echocardiographic parameters might be affected to its prognostic role. Furthermore, based on the experience of our center and the available evidence, we proposed an algorithm that might help clinicians distinguish from actual diastolic dysfunction from a normal diastolic pattern in HTx population.

## Introduction

The prevalence of people being affected by heart failure worldwide is incessantly increasing and is now over 60 million (1). Consensually, the ranks of those in advanced stages of the disease are expanding. Many treatment strategies are available for such patients with the common goal of supporting the mechanical function of the heart. Heart transplant (HTx) is recognized as the most effective destination therapy since the median survival time after transplantation exceeds 10 years nowadays (2). More than 5,000 HTx have been performed in 2015 worldwide, reaching the highest number since the technique’s introduction back in 1967 (2).

However, survival is still impaired by two groups of transplant-related complications: those dependent on immunosuppressive therapy (e.g., malignancies and infections) and those graft-specific, which include cardiac allograft vasculopathy (CAV) and acute and chronic graft rejection. In a growing donor organ shortage era, avoidance of graft failure as long as possible is of paramount importance.

In addition to invasive methods, such as coronary angiography, endomyocardial biopsy, and right heart catheterization, non-invasive methods have been widely used to track changes in post-transplant cardiac function, such as echocardiography, cardiac computed tomography particularly for CAV detection, and recently, cardiac magnetic resonance (3, 4). It is essential to understand the peculiar physiology of the transplanted heart and how it influences the traditional parameters used during the follow-up, in particular, echocardiographic ones. The transplanted heart is subjected to several changes, myocardial injury and ischemia time of the donor’s heart, denervation of the allograft, and peri-operative factors being the most implicated factors. Evidence suggests that both cardiac dimensions and functional parameters might be different from the general population (5), as shown in Table 1. Therefore, echocardiographic evaluation after HTx appears further complicated by the lack of standardized specific normal reference values for this population. In this context, one interesting aspect of HTx physiology is diastolic function. In fact, after surgery, the heart is subjected to several modifications, which tend to change over time, particularly the left atrium (LA), which is a major determinant of diastolic function, undergoing profound alterations.

**TABLE 1:** Reference values for diastolic parameters evaluated by echocardiography in heart transplant patients.

Reference values for diastolic parameters in HTx patients			
Echocardiographic parameter	Mean ± SD	Range (2.5^th^ to 97.5^th^ percentile)	95% CI of mean
E/A	1.8 ± 0.6	0.8–3.2	1.7–2.1
e’ (lateral) (cm/s)	8.0 ± 3.1	5.5–11.1	7.2–9.1
DT (m/s)	156 ± 31	101–120	146–165
E/e’ (lateral)	7.1 ± 3.0	3.1–14.7	6.4–8.4
LA volume/BSA (bicaval) (mL/m^2^)	41 ± 16	29–121	71–79
MV E (cm/s)	80 ± 21	50–120	75–87
LVGLS (%)	−16.5 ± 3.3	12–35	15–18

TR velocity and PALS are not reported since no study has determined them yet. Adjusted from Ingvarsson et al. (3). BSA, body surface area; CI, confidence interval; DT, deceleration time; LA, left atrium; LVGLS, left ventricular global longitudinal strain; MV, mitral valve; PALS, peak atrial longitudinal strain; SD, standard deviation; TR, tricuspid regurgitation.

The aim of this review is to attempt to provide current evidence on diastolic function in HTx starting from defining its different physiology and how the standard echocardiographic parameters might be affected to its prognostic role.

## Determinants of diastolic function in heart transplant patients

### Histological findings in diastolic dysfunction

From a histological standpoint, diastolic dysfunction is related to a substantial subversion of the extracellular matrix due to the presence of edema or fibrosis. Such tissue alterations determine the stiffening of myocardial walls and therefore alter lusitropic properties. In various pathological conditions, they occur before the overt manifestations of the disease, leaving space for pre-clinical detection. This could be true also for early identification of graft-specific complications since edema could be the result of acute graft rejection, while fibrosis may be the manifestation of both chronic graft rejection or CAV. However, recent evidence suggests that diastolic function might also be linked to microvascular density (6). Considering that, Daud et al. found that diastolic dysfunction in patients with severe CAV might be secondary to the loss and/or remodeling of microvasculature rather than a consequence of interstitial fibrosis (7).

### Cardiac allograft physiology

Graft physiology is considerably different from normal, as a consequence of various factors such as denervation, altered anatomy, and hemodynamic status of the recipient. First, the electrical impulse originates from the donor atrium and, at least in the first 6–12 months after transplant, it is not under any control of the recipient’s nervous system. Because of the reduced variability of heart rate, cardiac output is critically pre-load dependent in HTx. Second, because of the mismatch between the recipient and donor heart dimensions, the cardiac allograft is usually subject to enhanced mobility into the recipient cavity, enlarged by the dilated explanted heart, and clockwise rotated. Finally, atrial contribution to ventricular filling is reduced because of altered anatomy and function consequent to surgical anastomosis. Usually, HTx patients are characterized by restrictive physiology during the first period, probably because of inflammatory edema related to ischemic reperfusion injury, allograft ischemic time, surgery, and/or immune-mediated acute response. During follow-up, the diastolic pattern tends to improve after the first few weeks progressing to a non-restrictive filling pattern during the first year (8, 9). Nonetheless, in some patients, an abnormal diastolic filling can be identified many years after transplantation and this correlates with symptoms of heart failure and a history of acute rejection episodes (10).

### The effects of surgical techniques on diastolic function

**FIGURE 1:**
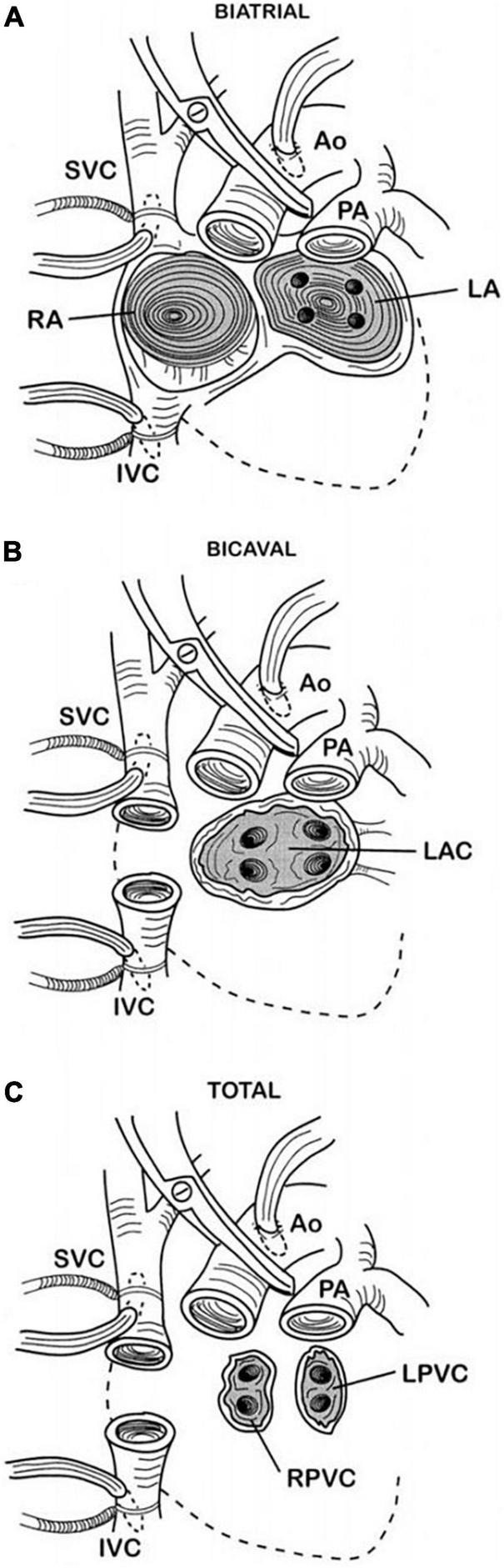
Surgical techniques for orthotopic heart transplant. Picture **(A)** shows the biatrial technique in which the anastomoses are at the mid-level of the left and right atria in addition to the aortic and pulmonary artery anastomoses. Picture **(B)** depicts the bicaval technique, the most commonly used nowadays, in which separate superior and inferior vena cava anastomoses are made instead of the right atrial anastomosis. Finally, picture **(C)** shows the total orthotopic heart transplant technique, which is a complete atrioventricular cardiac transplantation with separate cava and pulmonary vein anastomoses. Kindly readapted from Badano et al. (16). Dotted lines, original position of excised native heart; Ao, aorta; IVC, inferior vena cava; LA, left atrium; LAC, left atrial cuff; LPVC, left pulmonary vein cuff; PA, pulmonary artery; RA, right atrium; RPVC, right pulmonary vein cuff; SVC, superior vena cava.

The atrial function is variably altered in HTx patients according to the employed surgical technique. With the “biatrial technique,” in which the posterior cuffs of the recipient atria are left in place and attached to the donor atria, the atria result enlarged with an altered geometry, known as “snowman” configuration. In addition, impaired electrical impulse initiation (due to sinus node injury) or conduction could result in brady- or tachyarrhythmias, including atrial fibrillation (11). To overcome these limitations, two alternative techniques have been introduced over the years: the “bicaval technique” and the “total technique.” The former preserves the LA anastomosis but combines it with bicaval anastomosis, whereas the latter preserves the integrity of both atria but requires to anastomose both the inferior and superior vena cava and the pulmonary veins. However, the “total technique” is infrequently employed because it is technically demanding. The bicaval technique better preserves atrial anatomy and function compared to the biatrial technique (12, 13); therefore, it is the most widely chosen one. Figure 1 shows the different surgical techniques used in heart transplantation. A recent study showed that both LA and right atrial function, in particular the reservoir phase, are impaired in a population of HTx patients operated with the bicaval technique (14). Particularly, they found that LA reservoir function was more profoundly reduced in presence of a larger LA and increased LV filling pressures, whereas a reduced RA reservoir function was associated with a decreased RV longitudinal function. As mentioned earlier, both atria undergo profound alterations during HTx, even if the bicaval technique is used over the biatrial one, which almost inevitably ends with a certain degree of atrial fibrosis. The atrial reservoir function is significantly dependent not only on ventricular longitudinal function but also on the compliance of the atrium, which is strictly linked to its stiffness and relaxation properties. In particular, since the LA is the chamber mostly and more directly affected by the operation, the association between LA reservoir function and larger LA as well as higher LV filling pressure could be comprehended (14). On the other hand, due to a less extended structural change, it is reasonable to understand a closer correlation between RA function and right ventricular longitudinal function, which is the other major determinant in atrial reservoir function.

## Echocardiographic assessment of diastole

### Challenges in diastolic evaluation

Echocardiography represents the first-line imaging modality to assess diastolic function and it is the cornerstone exam in the follow-up of HTx patients. However, the evaluation of diastolic function in HTx is challenging since the most widely employed diastolic parameters are sensible to heart motion as well as acoustic angle. Therefore, it is unlikely that the usual cut-off values can be appropriately applied to HTx patients (15). For these reasons, when studying the diastolic function of a cardiac allograft, it is more important to record individual parameters’ variability over time instead of focusing on absolute values themselves. The basal echocardiographic assessment should be performed at least 6 months from surgery, since in earlier examinations, many parameters may be physiologically altered (16).

### Comprehensive diastolic assessment

According to the latest recommendations (15), at least four echocardiographic variables should always be assessed when evaluating LV diastolic function, including mitral annular e’ velocity, preferably both lateral and septal, average E/e’ ratio, LA volume index, and peak tricuspid regurgitation velocity. The analysis of mitral inflow velocities and mitral annular tissue Doppler is fundamental for estimating LA pressure, that is LV filling pressure, which in turn correlates better with pulmonary capillary wedge pressure (15). The application of tissue Doppler imaging (TDI) in the assessment of diastolic function improves the accuracy of the echocardiographic exam as Doppler parameters of transvalvular flow are load and heart rate dependent. Furthermore, mainly with the use of the biatrial technique, there is often atrial dissociation and variation in transmitral E and A waves’ velocities, limiting their application in the estimation of filling pressures (17). It is also true that TDI velocities may be affected by the exaggerated translation motion of the allograft (18). Additional traditional indexes that should be performed are represented by pulmonary vein velocities and those derived by speckle tracking echocardiography (STE), such as LA strain and LV global longitudinal strain (LV-GLS). Table 2 summarizes the limitations and characteristics of echocardiography-derived diastolic parameters in HTx.

**TABLE 2:** Advantages and disadvantages of echocardiography-derived diastolic parameters in heart transplant.

Echocardiographic diastolic parameters	Graft specific limitations and characteristics
Mitral inflow velocities (E wave velocity; A wave velocity; E/A ratio)	**Limitations:** Reduced atrial contribution to ventricular filling **Characteristics:** **1.** Restrictive filling pattern soon after cardiac transplantation: Shortened IVRT, high peak E wave velocity, shortened DT **2.** Non-restrictive pattern: Prolonged IVRT and DT, decreased transmitral early filling velocities. **3.** Reduced atrial contribution to LV filling, E/A could still be ≥2 even though LV filling pressures are low
TDI derived velocities (lateral and septal mitral annular e’ velocity)	**Limitations:** Exaggerated translation motion (mismatch recipient–donor, enhanced mobility), clockwise rotation of HTx **Characteristics:** Low mitral annular TDI velocities with an increasing trend over time, even though they are lower than general population 1 year after HTx
E/e’ ratio	**Limitations:** Exaggerated translation motion (mismatch recipient–donor, enhanced mobility), clockwise rotation of HTx which influence the measurement of TDI derived velocities **Characteristics:** Transmitral velocities corrected for the influence of relaxation; valuable index of LV filling pressures
LA size	**Characteristics:** Atrial enlargement without clear impact on function
Pulmonary veins velocities	**Limitations:** Not valuable index of LV filling pressures
Speckle tracking echocardiography (LA-PALS, LA-PACS, LV-GLS, LV-GCS, Ssr, Esr)	**Limitations:** Susceptible to image quality, low frame rate, which is problematic in higher heart rates as seen in denervated transplanted heart. **Characteristics:** Altered LA and LV parameters (PALS, PACS and GLS, GCS); strong association with LV filling pressures. Ssr and Esr and E/Esr ratio correlate well with LV end-diastolic pressure and detect myocardial dysfunction earlier than LV-GLS.

HTx, heart transplantation; LA, left atrium; LV, left ventricle; IVRT, isovolumetric relaxation time; DT, deceleration time; TDI, tissue Doppler Imaging; PALS, peak atrial longitudinal strain; PACS, peak atrial contraction strain; LV-GLS, left ventricular global longitudinal strain; LV-GCS, left ventricular global circumferential strain; SR, strain rate; Sst, peak systolic strain rate; Esr, early diastolic strain rate.

### Changes in diastolic function after surgery

Soon after cardiac transplantation, Doppler echocardiographic indexes of LV diastolic function are suggestive of elevated filling pressures. Particularly, iso-volumetric relaxation time (IVRT) is shortened and the transmitral inflow pattern shows an increase in E wave velocity and a shortening of deceleration time (DT) (19). The opening of the mitral valve occurs during the rapid ventricular pressure decline resulting in high peak early mitral flow velocity (E wave). Besides, the elevated LV filling pressure and the abrupt rise in early diastolic pressure explain the rapid deceleration of transmitral flow velocity with a shortened DT; also, LA pressure is increased and may contribute to the earlier opening of the mitral valve with a shortened IVRT (10). Moreover, it is common to find low TDI velocities at the mitral annular level, which tend to gradually increase over time, despite the fact that HTx values remain lower compared to the general population, even after 1 year (20). The elevation in filling pressure observed in the first month after surgery is due to the tendency of fluid accumulation due to a systemic inflammatory state and high doses of corticosteroids, in addition to the abovementioned reasons, such as inflammatory edema related to ischemic reperfusion injury, allograft ischemic time, surgery, and/or immune-mediated acute response. The restrictive diastolic pattern occurs irrespective of rejection status, as shown by studies assessing echocardiographic indexes on the day that endomyocardial biopsy was performed (8, 20), as well as independently of the surgical technique used (5) and clinical variables such as pre-operatory pulmonary pressure and the age of the donor’s heart (19). In very few cases, the restrictive physiology might be predominantly explained by prolonged donor organ ischemia (21).

**FIGURE 2:**
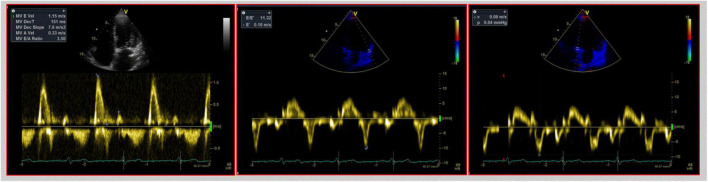
Echocardiographic assessment of diastolic function in heart transplant. This figure shows a mitral E/A ratio ≥2 with a deceleration time of E wave of 151 ms, thus indicating a possible restrictive filling pattern in a 3-year heart transplant patient. However, TDI analysis, shown in the middle and right pictures, shows normal e’ lateral and septal velocities, thus possibly excluding a restrictive filling pattern.

**FIGURE 3:**
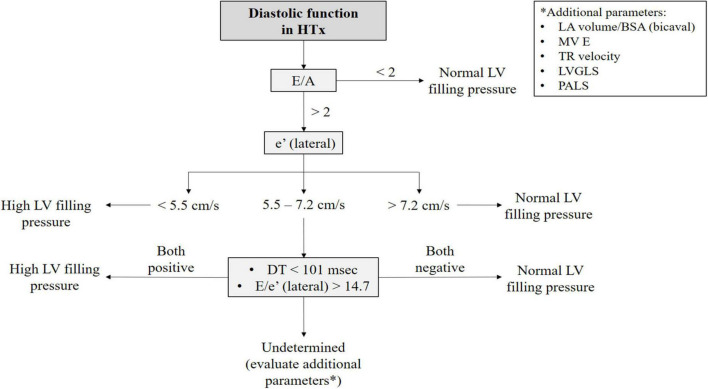
Proposed algorithm to evaluate diastolic function in heart transplant patients. The first step in the evaluation of diastolic function in HTx is assessing mitral inflow velocities. In the case of an E/A ratio <2 high LV filling pressure can be fairly excluded. On the other hand, if the E/A ratio >2, it is useful to evaluate TDI-derived velocities. However, when their values lie in a gray zone, DT and E/e’ ratio should be considered. If these latter two indexes together cannot exclude high LV filling pressure, additional parameters should be used, such as LAVi (in the case of bicaval technique), LA strain, and LV-GLS and TR velocity. This is the diagnostic algorithm proposed by our center, based on the values derived from the study by Ingvarsson et al. (5). BSA: body mass index; DT: deceleration time; HTx: heart transplantation; LA: left atrial; LAVI: left atrial volume index; LV: left ventricular; LVGLS: left ventricular global longitudinal strain; MV: mitral valve; PALS: peak atrial longitudinal strain; TR: tricuspid regurgitation.

As the diastolic function improves with time, a progression to a non-restrictive pattern is seen. IVRT and DT prolong and transmitral early filling velocities decrease (19). However, during follow-up, the mitral E/A ratio could still be ≥2, thus indicating a possible restrictive filling pattern, even though LV diastolic function may be normal. LA impairment caused by surgical intervention leads to a reduced atrial component to LV filling explaining the increased E/A ratio (Figure 2). Conversely, TD-derived diastolic velocities strongly correlate with altered relaxation and diastolic dysfunction. The ratio E/e’, combining TDI parameters with mitral inflow velocities, corrects transmitral velocities for the influence of relaxation and is a valuable index of LV filling pressures and diastolic dysfunction in both the biatrial (17) and bicaval techniques (22). Based on our experience and the available evidence on reference values in HTx patients, we proposed an algorithm that might help clinicians distinguish actual diastolic dysfunction with high LV filling pressure from normal LV filling pressure in the HTx population (Figure 3).

In particular, the proposed algorithm, shown in Figure 3, was created because of the limited application of each diastolic parameter alone. Cut-off values of each parameter were derived from the largest available prospective study by Ingvarsson et al. in a group of 124 clinically stable HTx patients (5), since standardized specific normal reference values for HTx patients are lacking. In particular, the assessment of TDI-derived velocities and DT might carry additional information on the diastolic function when the E/A ratio is above 2. In fact, according to the experience of our center, if these latter two indexes together point toward a restrictive filling pattern, the probability of diastolic dysfunction is high. Otherwise, additional parameters should be used to investigate diastolic function, as mentioned below.

### Additional echocardiographic parameters

#### Pulmonary veins pattern

In HTx, the anastomoses at the level of the pulmonary vein ostia interfere with pulmonary vein flow, except from the biatrial technique (23). In addition to that, because the contractility of the remnant recipient atrial tissue alters the various components of pulmonary veins flow, this variable is not valuable for assessing LV filling pressures, irrespective of the surgical technique used (15).

#### Left atrial size

A significant atrial enlargement is seen among HTx patients irrespective of surgical technique, although it is more pronounced with the biatrial one because of the remaining atrial roof from the recipient (5, 24). In a prospective study by Ingvarsson et al. (5), they reported the following reference values for atrial dimensions in a group of 124 stable HTx patients: left atrial volume (mL), 96 ± 47 in the biatrial group vs. 75 ± 23 in the bicaval (*p* < 0.001), and left atrial volume/BSA (mL/m^2^), 53 ± 23 vs. 39 ± 13 (*p* < 0.001). In patients operated on with the bicaval technique, atrial volume correlates only with allograft age instead (5). However, the impact of LA size on function is not completely clear as there is scarce evidence regarding the comparison of LA function between the two techniques (24, 25).

#### Left atrial strain

The LA acts as both a reserve and a conduit and also as an ancillary pump. In stable post-transplant patients, LA function is altered in all its functions regardless of the surgical technique as demonstrated in a study by Zhu et al. on 112 clinically well HT patients compared to healthy controls. In this study, functional comparison using STE showed a significant difference between HT patients and controls (PALS: 18.1 ± 5.6% vs. 44.2 ± 6.5% and PACS 4.4 ± 2.3% vs. 17.5 ± 4.7%; both *p* < 0.001) (26). In addition to a reduction in LA-PALS, STE showed a linear negative correlation between PALS and advanced recipient age, larger LA volumes, and worse LV systolic function measured by LV ejection fraction and LVGLS, suggesting that atrial function is altered not only due to surgery but also as a consequence of ventricular dysfunction (26). Also, PALS helps in the detection of diastolic dysfunction as LV end-diastolic pressure is the afterload on the LA during the reservoir phase (27). An association between increased ventricular filling pressures and reduced atrial strain has been observed (28). Furthermore, a recent study hinted at a possible role of LA-PALS in detecting ACR (29). Rodriguez-Diego et al. found a significant decrease in PALS in presence of any degree of ACR, even though significant inter-vendor strain reproducibility was reported (29). A possible explanation for this finding might be found in the role of PALS in detecting subtle diastolic changes related to ACR episodes, in which different grades of inflammation affect the myocardium.

#### Left ventricular longitudinal strain

Due to the aforementioned limitations of the traditional indices of diastolic function for the estimation of LV filling pressures in HTx, the performance of myocardial deformation analysis using STE to predict elevated pulmonary capillary wedge pressure in HTx has been studied. Strain and strain rate parameters such as GLS and GCS have stronger diagnostic performance than traditional parameters of diastolic dysfunction, such as E/e’ (30). Ingvarsson et al. found a reduction in LV-GLS in a group of HTx patients compared with reported normal values (mean LV-GLS −16 ± 3.3%; *p* < 0.001 and mean LV-GCS −22.9 ± 6.3%; *P* = NS) possibly due to surgical procedure and progressive remodeling including myocardial fibrosis and/or previous rejections (5, 31). LV-GLS and LV-GCS are strongly associated with LV filling pressures as there is a tight coupling of systolic and diastolic functions and also a rise in filling pressures increases wall tension resulting in depressed myocardial systolic deformation (30). Regarding longitudinal diastolic strain rate, defined as the rate of deformation in percent of strain per second during diastole (32), there is evidence that peak systolic (Ssr) and early diastolic (Esr) strain rate and the ratio of transmitral early filling velocity to early diastolic strain rate correlate well with LV end-diastolic pressure and pulmonary capillary wedge pressure, also tracking well the changes of these parameters with time thus detecting myocardial dysfunction earlier than LV-GLS (30).

## Prognostic implications of diastolic assessment

Graft failure due to acute cellular rejection is a common complication of HTx and the main cause of mortality in the first years after surgery (33), whereas extensive CAV is seldom seen as early as 1 year after surgery (34). The current gold standard method for diagnosing rejection is an endomyocardial biopsy (35) but other non-invasive imaging methods—of which echocardiography is the first line imaging modality—play an important role in assessing and monitoring allograft function (16). Acute graft rejection is categorized into acute cellular or antibody-mediated rejection (36, 37) and induces myocardial lymphocyte infiltration and edema manifested earlier by impaired LV filling and later by increased wall thickness and systolic dysfunction (38). Acute cellular rejection correlates with shortening of the IVRT and early mitral inflow DT, while changes in E and A wave velocities and E/A ratio have been less consistent (39). Variations in transmitral Doppler flow indices are also rather non-specific in detecting rejection as they are markedly influenced by other variables, such as heart rate, age, and loading conditions. Diastolic function assessed by transmitral Doppler diastolic indexes should allow the sensitive detection of acute rejection but their value is limited as they can be abnormal even in healthy patients (10). Nevertheless, diastolic dysfunction carries a prognostic value (40), and Doppler abnormalities in LV filling patterns have shown a return to baseline following episodes of rejection (41). TDI and relaxation velocities have been studied and results are not univocal, since, in some studies, it has been found that an association between decreased systolic and filling velocities and acute rejection (42, 43) is not confirmed by others (44). It can be said that TDI velocities are highly specific as they have a good negative predictive value, so rejection could be excluded in the presence of <10% reduction in diastolic mitral annular motion velocities (44, 45). The aforementioned markers of acute rejection are based on abnormalities in LV filling; speckle tracking-derived LV-GLS is a sensitive marker for the detection of sub-clinical regional systolic function abnormalities instead (46). Diastolic speckle tracking indexes can detect subclinical dysfunction during acute cellular rejection at an earlier stage than LV-GLS, particularly E/GDSRe, as it can detect functional alterations even in the context of normal E/e’ ratio (32). Finally, a completely normal echocardiographic examination provides a high negative predictive value for detecting acute graft rejection at endomyocardial biopsy while there is a significant correlation between the number of abnormal echocardiographic parameters and rejection grade (47).

Diastolic dysfunction carries a significant prognostic value also in chronic graft rejection, which is mainly determined by CAV and, in some patients, triggered by recurring immune responses against the graft resulting in replacement fibrosis and progressive deterioration of myocardial function, especially in patients with alloreactive antibodies. Histologically, CAV is a diffuse vasculopathy secondary to a fibroproliferative process initially resulting in concentric narrowing of both the large epicardial coronary arteries, the coronary veins, and the microcirculation, and later on, in focal luminal stenoses detectable with coronary angiography (16). Diastolic dysfunction is a key element in the grading of CAV; in fact, according to the latest classification of CAV by ISHLT, severe CAV is defined in presence of visual coronary angiographic stenosis and evidence of graft dysfunction such as reduced LVEF end/or restrictive filling pattern (48). It follows that diastolic function is markedly impaired in patients with severe CAV, generally resulting in restrictive cardiac physiology, defined as symptomatic heart failure with an echocardiographic E/A velocity ratio >2, shortened IVRT (<60 ms), shortened DT (<150 ms), or restrictive hemodynamic values (48). Instead, in patients with severe CAV, LVEF is typically preserved, even though it tends to show lower values compared with mild CAV (7); nevertheless, the occurrence of a reduction in LVEF years later after HTx should prompt other investigations to exclude CAV. Earlier detection of ventricular dysfunction may be investigated with TDI-derived velocities and STE with CAV patients presenting with augmented duration and reduced amplitude of TDI-myocardial velocities (39) and the reduced absolute value of LV-GLS (49, 50). Evidence suggests that the key histopathologic finding in CAV-related diastolic dysfunction is increased capillary wall thickness and reduced capillary density, rather than interstitial fibrosis, which has similar extent in severe CAV and non-significant CAV patients (7, 51). Furthermore, the restrictive physiology carries a prognostic significance in CAV patients, as it has been related to a lower 5-year survival (52).

In conclusion, no single diastolic parameter reliably predicts graft-specific complications (47), and a comprehensive echocardiographic evaluation of diastolic function should be performed at every follow-up visit, particularly focusing not on absolute values of the various parameter but rather on their variation over time. Performing an appropriate baseline echocardiographic exam is fundamental for this purpose.

## Conclusion

The assessment of LV diastolic function is considered an integral part of the clinical evaluation of HTx patients. It carries a relevant prognostic value in the follow-up, helping in the early detection of possible complications such as rejections. However, its assessment requires several considerations due to the profound alterations that the transplanted heart undergoes, especially LA which plays a key role in defining diastolic function.

## Author contributions

CS, CF, FL, and MB participated in the writing the manuscript. GM and ML provided images and tables as well as a revision of the manuscript, whereas SV, FD’A, MF, and MC critically revised the manuscript. All authors contributed to the article and approved the submitted version.

## References

[1] James SL Abate D Abate KH Abay SM Abbafati C Abbasi N Global, regional, and national incidence, prevalence, and years lived with disability for 354 Diseases and Injuries for 195 countries and territories, 1990-2017: a systematic analysis for the Global Burden of Disease Study 2017. *Lancet.* (2018) 392:1789–858. 30496104 10.1016/S0140-6736(18)32279-7 PMC6227754

[2] Lund LH Khush KK Cherikh WS Goldfarb S Kucheryavaya AY Levvey BJ The registry of the international society for heart and lung transplantation: thirty-fourth adult heart transplantation report—2017; Focus theme: allograft ischemic time. *J Heart Lung Transpl.* (2017) 36:1037–46. 10.1016/j.healun.2017.07.019 28779893

[3] Olymbios M Kwiecinski J Berman DS Kobashigawa JA. Imaging in heart transplant patients. *JACC Cardiovasc Imaging.* (2018) 11:1514–30. 10.1016/j.jcmg.2018.06.019 30286911

[4] Salvo G Siblini G Issa Z Mohammed H Abu Hazeem A Pergola V Left ventricular mechanics in patients with abnormal origin of the left main coronary artery from the pulmonary trunk late after successful repair. *Di Cardiol.* (2017) 136:71–6. 10.1159/000447961 27562944

[5] Ingvarsson A Evaldsson AW Waktare J Nilsson J Smith GJ Stagmo M Normal reference ranges for transthoracic echocardiography following heart transplantation. *J Am Soc Echocardiogr.* (2018) 31:349–60. 10.1016/j.echo.2017.11.003 29275986

[6] Lavine KJ Sintek M Novak E Ewald G Geltman E Joseph S Coronary collaterals predict improved survival and allograft function in patients with coronary allograft vasculopathy. *Circ Heart Fail.* (2013) 6:773–84. 10.1161/CIRCHEARTFAILURE.113.000277 23709657 PMC3863089

[7] Daud A Xu D Revelo MP Drakos SG Dranow E Stoddard G Microvascular loss and diastolic dysfunction in severe symptomatic cardiac allograft vasculopathy. *Circ Heart Fail.* (2018) 11:e004759. 10.1161/CIRCHEARTFAILURE.117.004759 30354559

[8] Tallaj JA Kirklin JK Brown RN Rayburn BK Bourge RC Benza RL Post-heart transplant diastolic dysfunction is a risk factor for mortality. *J Am Coll Cardiol.* (2007) 50:1064–9. 10.1016/j.jacc.2007.06.007 17825716

[9] Greenberg ML Uretsky BF Reddy PS Bernstein RL Griffith BP Hardesty RL Long-term hemodynamic follow-up of cardiac transplant patients treated with cyclosporine and prednisone. *Circulation.* (1985) 71:487–94. 10.1161/01.CIR.71.3.487 3882266

[10] Valantine HA Appleton CP Hatle LK Hunt SA Billingham ME Shumway NE A hemodynamic and Doppler echocardiographic study of ventricular function in long-term cardiac allograft recipients. Etiology and prognosis of restrictive-constrictive physiology. *Circulation.* (1989) 79:66–75. 10.1161/01.cir.79.1.66 2642757

[11] Chokesuwattanaskul R Bathini T Thongprayoon C Preechawat S O’Corragain OA Pachariyanon P Atrial fibrillation following heart transplantation: a systematic review and meta-analysis of observational studies. *J Evid Based Med.* (2018) 11:261–71. 10.1111/jebm.12323 30444058

[12] Traversi E Pozzoli M Grande A Forni G Assandri J Viganò M The bicaval anastomosis technique for orthotopic heart transplantation yields better atrial function than the standard technique: an echocardiographic automatic boundary detection study. *J Heart Lung Transplant.* (1998) 17:1065–74. 9855445

[13] Beniaminovitz A Savoia MT Oz M Galantowicz M Tullio MRD Homma S Improved atrial function in bicaval versus standard orthotopic techniques in cardiac transplantation. *Am J Cardiol.* (1997) 80:1631–5. 10.1016/S0002-9149(97)00756-X 9416956

[14] Bech-Hanssen O Pergola V Al-Admawi M Fadel BM Di Salvo G. Atrial function in heart transplant recipients operated with the bicaval technique. *Scand Cardiovasc J.* (2016) 50:42–51. 10.3109/14017431.2015.1091946 26467003

[15] Nagueh SF Smiseth OA Appleton CP Byrd BF Dokainish H Edvardsen T Recommendations for the evaluation of left ventricular diastolic function by echocardiography: an update from the American society of echocardiography and the European association of cardiovascular imaging. *J Am Soc Echocardiogr.* (2016) 29:277–314. 10.1016/j.echo.2016.01.011 27037982

[16] Badano LP Miglioranza MH Edvardsen T Colafranceschi AS Muraru D Bacal F European association of cardiovascular imaging/cardiovascular imaging department of the Brazilian society of cardiology recommendations for the use of cardiac imaging to assess and follow patients after heart transplantation. *Eur Heart J Cardiovasc Imaging.* (2015) 16:919–48. 10.1093/ehjci/jev139 26139361

[17] Sundereswaran L Nagueh SF Vardan S Middleton KJ Zoghbi WA Quiñones MA Estimation of left and right ventricular filling pressures after heart transplantation by tissue Doppler imaging. *Am J Cardiol.* (1998) 82:352–7. 10.1016/S0002-9149(98)00346-4 9708666

[18] Aranda JM Weston MW Puleo JA Fontanet HL. Effect of loading conditions on myocardial relaxation velocities determined by Doppler tissue imaging in heart transplant recipients. *J Heart Lung Transplant.* (1998) 17:693–7. 9703234

[19] StGoar FG Gibbons R Schnittger I Valantine HA Popp RL. Left ventricular diastolic function. Doppler echocardiographic changes soon after cardiac transplantation. *Circulation.* (1990) 82:872–8. 10.1161/01.cir.82.3.872 2394008

[20] Goland S Siegel RJ Burton K De Robertis MA Rafique A Schwarz E Changes in left and right ventricular function of donor hearts during the first year after heart transplantation. *Heart.* (2011) 97:1681–6. 10.1136/hrt.2010.220871 21586422

[21] Young JB Leon CA Short HD Noon GP Lawrence EC Whisennand HH Evolution of hemodynamics after orthotopic heart and heart-lung transplantation: early restrictive patterns persisting in occult fashion. *J Heart Transplant.* (1987) 6:34–43. 3112344

[22] Broch K Al-Ani A Gude E Gullestad L Aakhus S. Echocardiographic evaluation of left ventricular filling pressure in heart transplant recipients. *Scand Cardiovasc J.* (2014) 48:349–56. 10.3109/14017431.2014.981579 25414078

[23] Richards DR Gilliland Y Bernal JA Smart FW Stapleton DD Ventura HO Mitral inflow and pulmonary venous Doppler measurements do not predict pulmonary capillary wedge pressure in heart transplant recipients. *Am Heart J.* (1998) 135:641–6. 10.1016/S0002-8703(98)70280-7 9539480

[24] Dell’Aquila AM Mastrobuoni S Bastarrika G Praschker BL Agüero PA Castaño S Bicaval versus standard technique in orthotopic heart transplant: assessment of atrial performance at magnetic resonance and transthoracic echocardiography. *Interact Cardiovasc Thorac Surg.* (2012) 14:457–62. 10.1093/icvts/ivr084 22217865 PMC3309807

[25] Bech-Hanssen O Al-Habeeb W Ahmed W Di Salvo G Pergola V Al-Admawi M Echocardiography detects elevated left ventricular filling pressures in heart transplant recipients. *Echocardiography.* (2015) 32:411–9. 10.1111/echo.12683 24995376

[26] Zhu S Xie Y Qiao W Tian F Sun W Wang Y Impaired left atrial function in clinically well heart transplant patients. *Int J Cardiovasc Imaging.* (2021) 37:1937–45. 10.1007/s10554-021-02177-4 33620609

[27] Morris DA Belyavskiy E Aravind-Kumar R Kropf M Frydas A Braunauer K Potential usefulness and clinical relevance of adding left atrial strain to left atrial volume index in the detection of left ventricular diastolic dysfunction. *JACC.* (2018) 11:1405–15. 10.1016/j.jcmg.2017.07.029 29153567

[28] Singh A Addetia K Maffessanti F Mor-Avi V Lang RM. LA strain for categorization of LV diastolic dysfunction. *JACC Cardiovasc Imaging.* (2017) 10:735–43. 10.1016/j.jcmg.2016.08.014 28017389 PMC5741456

[29] Rodríguez-Diego S Ruiz-Ortiz M Delgado-Ortega M Weinsaft JW Sánchez-Fernández JJ Ortega-Salas R The role of left atrial longitudinal strain in the diagnosis of acute cellular rejection in heart transplant recipients. *J Clin Med.* (2022) 11:4987. 10.3390/jcm11174987 36078920 PMC9456609

[30] Colak A Muderrisoglu H Pirat B Eroglu S Aydinalp A Sezgin A Longitudinal strain and strain rate for estimating left ventricular filling pressure in heart transplant recipients. *Am J Cardiol.* (2020) 137:63–70. 10.1016/j.amjcard.2020.09.037 32998008

[31] Yingchoncharoen T Agarwal S Popović ZB Marwick TH. Normal ranges of left ventricular strain: a meta-analysis. *J Am Soc Echocardiogr.* (2013) 26:185–91. 10.1016/j.echo.2012.10.008 23218891

[32] Chamberlain R Scalia GM Shiino K Platts DG Sabapathy S Chan J. Diastolic strain imaging: a new non-invasive tool to detect subclinical myocardial dysfunction in early cardiac allograft rejection. *Int J Cardiovasc Imaging.* (2020) 36:317–23. 10.1007/s10554-019-01725-3 31720881

[33] Stehlik J Edwards LB Kucheryavaya AY Aurora P Christie JD Kirk R The registry of the international society for heart and lung transplantation: twenty-seventh official adult heart transplant report–2010. *J Heart Lung Transplant.* (2010) 29:1089–103. 10.1016/j.healun.2010.08.007 20870164

[34] Prada-Delgado O Estévez-Loureiro R Paniagua-Martín MJ López-Sainz A Crespo-Leiro MG. Prevalence and prognostic value of cardiac allograft vasculopathy 1 year after heart transplantation according to the ISHLT recommended nomenclature. *J Heart Lung Transplant.* (2012) 31:332–3. 10.1016/j.healun.2011.12.006 22333404

[35] Costanzo MR Dipchand A Starling R Anderson A Chan M Desai S The International Society of Heart and Lung Transplantation Guidelines for the care of heart transplant recipients. *J Heart Lung Transplant.* (2010) 29:914–56. 20643330 10.1016/j.healun.2010.05.034

[36] Stewart S Winters GL Fishbein MC Tazelaar HD Kobashigawa J Abrams J Revision of the 1990 working formulation for the standardization of nomenclature in the diagnosis of heart rejection. *J Heart Lung Transplant.* (2005) 24:1710–20. 10.1016/j.healun.2005.03.019 16297770

[37] Berry GJ Angelini A Burke MM Bruneval P Fishbein MC Hammond E The ISHLT working formulation for pathologic diagnosis of antibody-mediated rejection in heart transplantation: evolution and current status (2005-2011). *J Heart Lung Transplant.* (2011) 30:601–11. 10.1016/j.healun.2011.02.015 21555100

[38] Amende I Simon R Seegers A Daniel W Heublein B Hetzer R Diastolic dysfunction during acute cardiac allograft rejection. *Circulation.* (1990) 81(2 Suppl):III66–70. 2297883

[39] Thorn EM de Filippi CR. Echocardiography in the cardiac transplant recipient. *Heart Fail Clin.* (2007) 3:51–67. 17545009 10.1016/j.hfc.2007.02.008

[40] Ross HJ Gullestad L Hunt SA Tovey DA Puryear JB McMillan A Early Doppler echocardiographic dysfunction is associated with an increased mortality after orthotopic cardiac transplantation. *Circulation.* (1996) 94(9 Suppl):II289–93. 8901762

[41] Seacord LM Miller LW Pennington DG McBride LR Kern MJ. Reversal of constrictive/restrictive physiology with treatment of allograft rejection. *Am Heart J.* (1990) 120:455–9. 10.1016/0002-8703(90)90103-5 2382630

[42] Dandel M Hummel M Müller J Wellnhofer E Meyer R Solowjowa N Reliability of tissue Doppler wall motion monitoring after heart transplantation for replacement of invasive routine screenings by optimally timed cardiac biopsies and catheterizations. *Circulation.* (2001) 104(12 Suppl 1):I184–91. 10.1161/hc37t1.094855 11568053

[43] Puleo JA Aranda JM Weston MW Cintrón G French M Clark L Noninvasive detection of allograft rejection in heart transplant recipients by use of Doppler tissue imaging. *J Heart Lung Transplant.* (1998) 17:176–84. 9513856

[44] Stengel SM Allemann Y Zimmerli M Lipp E Kucher N Mohacsi P Doppler tissue imaging for assessing left ventricular diastolic dysfunction in heart transplant rejection. *Heart.* (2001) 86:432–7. 10.1136/heart.86.4.432 11559685 PMC1729918

[45] Dandel M Hetzer R. The use of echocardiography post heart transplantation. *Expert Rev Cardiovasc Ther.* (2016) 14:1161–75. 10.1080/14779072.2016.1214574 27428864

[46] Marciniak A Eroglu E Marciniak M Sirbu C Herbots L Droogne W The potential clinical role of ultrasonic strain and strain rate imaging in diagnosing acute rejection after heart transplantation. *Eur J Echocardiogr.* (2007) 8:213–21. 10.1016/j.euje.2006.03.014 16716752

[47] Mena C Wencker D Krumholz HM McNamara RL. Detection of heart transplant rejection in adults by echocardiographic diastolic indices: a systematic review of the literature. *J Am Soc Echocardiogr.* (2006) 19:1295–300. 10.1016/j.echo.2006.04.029 17000376

[48] Mehra MR Crespo-Leiro MG Dipchand A Ensminger SM Hiemann NE Kobashigawa JA International Society for Heart and Lung Transplantation working formulation of a standardized nomenclature for cardiac allograft vasculopathy-2010. *J Heart Lung Transplant.* (2010) 29:717–27. 10.1016/j.healun.2010.05.017 20620917

[49] Sciaccaluga C Mandoli GE Sisti N Natali MB Ibrahim A Menci D Detection of cardiac allograft vasculopathy by multi-layer left ventricular longitudinal strain in heart transplant recipients. *Int J Cardiovasc Imaging.* (2021) 37:1621–8. 10.1007/s10554-020-02147-2 33442856

[50] Clemmensen TS Løgstrup BB Eiskjær H Poulsen SH. Evaluation of longitudinal myocardial deformation by 2-dimensional speckle-tracking echocardiography in heart transplant recipients: relation to coronary allograft vasculopathy. *J Heart Lung Transplant.* (2015) 34:195–203. 10.1016/j.healun.2014.07.008 25108908

[51] Mohammed SF Hussain S Mirzoyev SA Edwards WD Maleszewski JJ Redfield MM. Coronary microvascular rarefaction and myocardial fibrosis in heart failure with preserved ejection fraction. *Circulation.* (2015) 131:550–9. 25552356 10.1161/CIRCULATIONAHA.114.009625 PMC4324362

[52] Itagaki BK Kobashigawa JA Wu GW Kawano MA Kittleson MM Fishbein MC Widespread fibrosis of myocardial and adjacent tissues causing restrictive cardiac physiology in patients needing re-do heart transplant. *J Heart Lung Transplant.* (2007) 26:S138.

